# A Study on the Machinability of Steels and Alloys to Develop Recommendations for Setting Tool Performance Characteristics and Belt Grinding Modes

**DOI:** 10.3390/ma13183978

**Published:** 2020-09-08

**Authors:** Nelli Vladimirovna Syreyshchikova, Viktor Ivanovich Guzeev, Dmitrii Valerievich Ardashev, Danil Yurievich Pimenov, Karali Patra, Wojciech Kapłonek, Krzysztof Nadolny

**Affiliations:** 1 Department of Automated Mechanical Engineering, South Ural State University, Lenin Prosp. 76, 454080 Chelyabinsk, Russia; snv.ktn@mail.ru (N.V.S.); gvi174@yandex.ru (V.I.G.); ardashevdv@susu.ru (D.V.A.); kpatra@iitp.ac.in (K.P.); 2 Department of Mechanical Engineering, Indian Institute of Technology Patna, Patna 801103, India; 3 Department of Production Engineering, Faculty of Mechanical Engineering, Koszalin University of Technology, Racławicka 15-17, 75-620 Koszalin, Poland; wojciech.kaplonek@tu.koszalin.pl (W.K.); krzysztof.nadolny@tu.koszalin.pl (K.N.)

**Keywords:** belt grinding, machining, machinability, classification, machinability groups, design, recommendations

## Abstract

This article presents a methodology for designing belt grinding operations with grinding and lapping machines. It provides the results of a study on the machinability of various steels and alloys with belt grinding, which are then classified according to an indicator that we have developed. Namely, cast aluminum alloys, structural alloy steels, structural carbon steels, corrosion-resistant and heat-resistant stainless steels, and heat-resistant nickel alloys have been investigated. The machinability index is the ratio of the performance indicators of the grinding belt and the depth of cut to the indicators of grade 45 structural carbon steels (similar to steel AISI 1045) and similar steels and alloys. The performance indicators of the grinding belt are chosen from a set of calculated and estimated indicators. Experimentally determining the dependences of the performance indicators on the belt grinding modes and conditions, taking into account the established levels of machinability, allowed us to develop recommendations for designing belt grinding operations with grinding and lapping machines. The proposed methodology for designing belt grinding operations guarantees optimal performance and ensures that the necessary quality of the machinable surfaces is achieved. At the same time, it takes into account variable machining conditions, which change within specified limits.

## 1. Introduction

Grinding and polishing with flexible-base abrasive tools are widely used processes in the aerospace, automotive, shipbuilding, bearing, tool, wood pulp, and paper industries [1,2,3,4]. During rough machining, these technological processes ensure the removal of significant stock allowances (up to 400 mm^3^/mm·s) [5,6]. During final and finish machining they ensure that small surface roughness areas *Ra* (the arithmetic mean deviation of the assessed profile) are removed (up to 0.04–0.02 µm) [7,8] with a high degree of accuracy (up to 0.01–0.10 mm), depending on the grinding pattern [9,10] and the quality of the final machining (residual stresses in the surface layer of up to 60·10^5^ Pa at a depth of about 0.01–0.02 mm) [11,12,13,14].

Flexible-base abrasive tools can be used to machine round outer and inner surfaces, flat surfaces, and complex (including curved) surfaces of workpieces [15,16,17], including large castings, forgings, sheets, and strips; long-length pipes of various diameters, cam shafts and crankshafts, small workpieces, bearings, and rollers; and household items, including knives, forks, and spoons [18,19,20,21].

Due to the tools’ features, grinding and polishing with flexible-base abrasive tools have become widespread processes that can compete with grinding with abrasive wheel disks [22,23,24,25,26]. In some cases, these processes can be alternatives to the use of an abrasive water jet [27,28,29].

To expand the field of application for machining with flexible-base abrasive tools, the following steps are necessary for their optimization and in order to set the rational machining modes and scientifically grounded rate:

- Studying the basic laws of these processes;
- Identifying the mechanism of interaction between the tool and the workpiece;
- Developing a methodology for choosing the main parameters of the processes.

These problems are solved by taking into account their complexity and diversity. These issues are at the junction of various fields of knowledge, including engineering techniques, material science, organic and inorganic chemistry, construction engineering, physics, and mathematics.

### 1.1. Problem Statement

Belt grinding occupies a special place in abrasive machining. In terms of kinematics, dynamics, and their accompanying physical phenomena, this type of machining occupies an intermediate position between grinding with “rough” wheels (i.e., nearly undeformable) and machining with loose abrasive grains [30,31,32].

There are three basic grinding patterns: rigidly-mounted grains on an abrasive wheel disk; grains fixed on a flexible base; loose grains [33,34]. Out of these, the first pattern is the most studied and mastered. All grinding patterns have many common elements, as well as their own features [35,36].

It has been established that during grinding with an abrasive wheel disk, there is almost no elastic compression of grains in the process and the cutting depth is determined by the size of the free portion of the grain protruding from the adhesive. The grinding mechanism is different when using an abrasive belt because the belt becomes deformed [13,14,37,38]. During cutting, only some vertices of the surface profile of the flexible-base abrasive tool come into contact with the machinable surface. With “rigid” mounting of abrasive grains, the number of cutting grains is 10–15% of the total nominal number. During grinding with an abrasive belt, the number of active grains increases [13,39,40,41] due to the elastic properties of the base. With the appearance of elastic deformation and under the influence of heat generation, the adhesive bond undergoes elastic movement (at the molecular level). The moving molecules of the bond carry overloaded abrasive grains. In this case, elastic movement of the molecules of the cloth fibers soaked with the coolant (if used) can also occur. Under the action of the radial component of the cutting force, the cloth of the belt is compressed and the abrasive grains, which are contained in an elastic medium of glue and cloth, acquire some mobility. As a result, a larger number of grains could be involved in the process. When contact is lost with the workpiece, the belt becomes unloaded, which is accompanied by oscillation of the grains, then after decay the system returns to the initial state. Grains that are weakly fixed or poorly oriented in the bond crumble due to the destruction of the bond under the influence of external forces [39,42,43,44].

The nature of abrasive belt deformations during grinding [14,37] indicates that the abrasive grains of the belt are loaded more uniformly in the contact zone and that the cutting depth of each of grain will be more stable than when grinding; for example, with wheel disks in a ceramic bond [45,46]. Heat is distributed more uniformly, which contributes to the formation of a more uniform surface layer, a decrease in temperature in the cutting zone, and to the values of residual stresses [47,48].

In addition, according to the works of Maslov [49], Reznikov et al. [50], Shal’nov [51], Lur’e and Gichan [52], Lur’e [53], and other researchers, it has been established that the bond of a coated abrasive, having a low metal friction coefficient and not participating in the dispersion of metal, also contributes to a significant reduction in heat generation, friction work, and power consumed during grinding.

Out of the whole variety of abrasive products, coated abrasives (CA) account for 39, and grinding operations based on their application are used in various industries at all stages of machining operations. Most coated abrasives (95%) are made of sandpaper, while the vast majority (63%) are products made of emery cloth [54,55,56,57].

The market economy trend of improving the quality of products leads to a constant increase in the share of abrasive belt grinding operations out of the total volume of machining operations. Therefore, a relevant task needed to increase the productivity of operations with the use of abrasive belts is providing high requirements for the quality of the machinable workpiece surfaces. Analysis of the effectiveness of using grinding belts showed that the costs of machining parts frequently fluctuate due to irrational characteristics of cutting tools and modes in homogenous operations being set, with a tight annual production program.

One of the most promising directions for improving the efficiency of abrasive belt grinding operations is developing technically sound recommendations for choosing the characteristics of cutting tools and modes. This can be achieved through thorough analysis and by determining the relationships between tool performance and the cutting conditions of steels and alloys with different levels of machinability. It is important to determine recommendations for cutting modes during abrasive belt grinding with grinding and lapping machines, and this paper deals with this topic.

### 1.2. Tasks and Objectives

**The objective of the work** is to increase the performance rate of machining steels and alloys with abrasive cloth belts based on elaboration of the recommendations for the design of belt grinding operations.

The main tasks are:

- To determine the dependences of the performance indicators of the grinding belt on the type of material being machined;
- To develop an indicator for the machinability of materials with a grinding belt;
- To classify groups of materials machined by grinding belts based on quantitative assessment of their machinability;
- To develop recommendations for the use of tools for the belt grinding of materials with various levels of machinability with grinding and lapping machines.

## 2. Materials and Methods

### 2.1. Theoretical Provisions of the Belt Grinding Process

The importance and relevance of the objective is determined by the significant volume of grinding belt usage, which accounts for about 77% of all manufactured coated abrasives, with a rather wide range of characteristics (about 20,000), as well as a variety of operations and uses in the most important industries [54,55,56].

**Figure 1:**
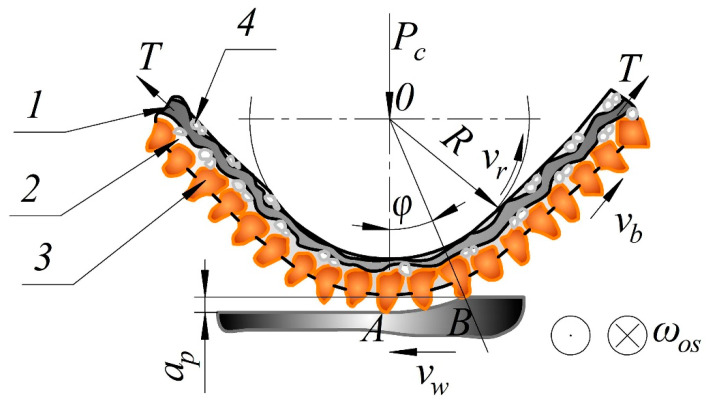
Pattern of flat belt grinding with longitudinal feed and vertical oscillation: 1—cloth backing; 2—bond; 3—grain; 4—coupling agent; φ—contact angle; ∪AB—contact arc length; *v_b_*—belt speed; *v_w_*—workpiece speed; *v_r_*—roller speed; *w_os_*—vertical oscillation frequency; *R*—roller radius; *P_c_*—clamping force; *T*—belt tension force; *a_p_*—cutting depth.

The Department of Automated Mechanical Engineering of the South Ural State University studied the metal belt grinding operations. Figure 1 shows a pattern of flat belt grinding with a longitudinal feed and vertical oscillation. The results of the study showed that the grinding modes used in production and tool performance characteristics are often random in nature.

For example, sandpapers with the same characteristics (made of normal aluminum oxide with fine-grained glue (up to P100)) are used to machine various materials and in different modes:

- For deburring pads during flat grinding of 10KP carbon steel (similar to AISI 1010) in the production of metal fittings;
- For external circular grinding of cylinder rods made of 35KH carbon steel (similar to AISI 5132) in the automotive industry;
- For external circular grinding of bearings made of wear-resistant SHKH15 steel (similar to alloy steel 52100) in the bearing industry [5,41,57].

The belt characteristics by type of operation (finishing, rough), i.e., by the size of the stock allowance and the roughness requirements, are applied unsystematically. For example, the 14A16C belt is used during flat grinding of stainless steel with a stock removal allowance of 1.0 mm to achieve a roughness of 1.25 μm. When machining cutlery, a larger grain 14A25K abrasive belt is used to remove a stock allowance that is 2 times smaller (0.5 mm) and to achieve the same roughness. In other production, when machining similar products, a belt with 2–4-fold smaller grain sizes (14A4–8 SFG) is used to remove the stock allowance of 0.7 mm and to achieve the same surface roughness. As a result, during operation there is significant excess consumption of the tool and it is used with low efficiency.

It has been determined that the difference in the performance of CA grinding of parts similar in size, accuracy, and surface quality, but made of steels and alloys of various grades and with all other things being equal, is preconditioned by differences in grinding machinability.

To create a justified system of grinding belt settings when machining various steels and alloys and to develop appropriate recommendations, we need to grade materials by their belt grinding machinability. The dependences of the performance indicators of the grinding belt on the type of material being machined were determined by empirical calculation based on the theoretical principles of the grinding process [58,59,60]. The essence of these provisions is as follows. The machinability of the same steels and alloys is determined by the physical relationship between the relative performance indicators characterizing the performance of the process and the mechanical properties of the materials being machined. The latter are determined by the intensity of stresses in the temperature–speed range of grinding deformations, as well as the geometry and blunting degree of the tool grains. The performance of the grinding process is determined by the analytical model of the estimated depth of cut [58]. The model takes into account the influence of the radial force at the cutting grain, the intensity of stresses in the shear zone of the material being machined, the geometry of the cutting portion of abrasive grains, and the nature of metal yielding in the deformation zone. It also takes into account the small turning angle of the grain during grinding under the influence of the cutting forces, the geometric parameters of the relief of the tool’s working surface. and the properties of the bond change during machining.

**Figure 2:**
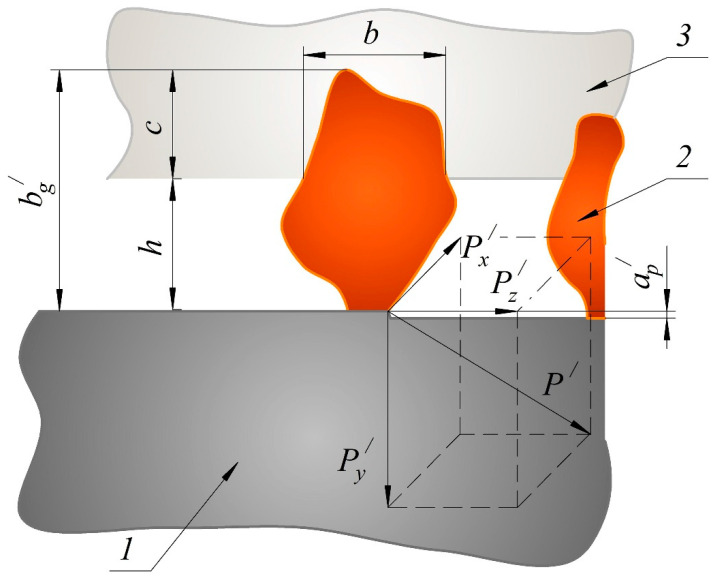
The scheme of the cutting grain of a grinding tape: 1—workpiece; 2—grain; 3—bond; *h*—grain protrusion from the bond; *c*—grain bonding; $b_{g}^{/}$—height of a single grain; *b*—grain width; $a_{p}^{/}$—depth of cut for one cutting grain; $P_{x}^{/}$—axial component of the cutting force acting on one grain; $P_{y}^{/}$—radial component of the cutting force acting on one grain; $P_{z}^{/}$—tangential component of the cutting force acting on one grain; *P^/^*—cutting force acting on one grain.

According to many researchers (Korchak [58], Maslov [49], Reznikov and Shelipanov [61]), the depth of cut for one cutting grain ($a_{p}^{/}$) largely determines the load on the grinding grain and the behavior of the grinding process. Therefore, the equation for determining the depth of cut $a_{p}^{/}$ can be considered as the main grinding equation. Figure 2 shows a diagram for determining the depth of cut made with the cutting grain of a grinding belt.

In our opinion, the physical essence of the grain grinding process expressed through the formula for the depth of cut $a_{p}^{/}$ is most reflected in the analytical model proposed by Korchak (for a conventional depth of cut $a_{p}^{/}$), which is fully applicable to any kind of abrasive tool. The model establishes the relationship between the depth of cut made with one grain and the mechanical properties of the materials being machined. The latter are determined by the intensity of stresses in the temperature–speed range of grinding deformations, as well as the geometry and the blunting degree of the tool grains [58]:

(1) $$a_{p}^{/}=\frac{P_{y}}{K_{1}\cdot \sigma_{i}}-K_{2}\cdot V_{B}\left(\tau \right)$$

where $a_{p}^{/}$ is the conditional depth of cut for a radial force $P_{y}$ per 1 mm of the cutting edge of the grain; $\sigma_{i}$ is the stress intensity in the shear zone of the material being machined (the stress intensity is a function of the strain intensity, ε, the strain rate, $\dot{\varepsilon }$, and the temperature, $T^{0}$, of the material: $\sigma_{i}=f\left(\varepsilon ,\dot{\varepsilon },T^{0}\right)$) [62,63,64]; $K_{1}$ and $K_{2}$ are the coefficients accounting for the geometry of the cutting part of the abrasive grains and the nature of metal yielding in the deformation zone; $V_{B}\left(\tau \right)$ is the grain blunting area, which changes during machining (flank wear).

The data on the stress intensity and the values of the coefficients $K_{1}$ and $K_{2}$ in Formula (1) are taken from the works of the Samara school [12,65]. They were obtained for steels and alloys of different grades during grinding with a single grain, and take into account a number of belt grinding features (using the cut shrinkage coefficients of various materials at belt grinding temperatures). Additionally, they are adjusted to consider the actual contact area of the belt with the workpiece, which changes during machining and is related to the rigidity of the belt ($V_{B}$) and the orientation of the grains.

Thus, we determined the values included in the formula for calculating the depth of cut:

(2) $$a_{p}^{/}=2.2027\cdot \frac{P_{y}}{\sigma_{i}}-0.0042\cdot V_{B}$$

In Formula (2), the size of the grain blunting area $V_{B}$ can be represented analytically based on the calculated dependence proposed by Pirozerskaya for the angle of the cutting grain deviation (β) from the vertical position during grinding [66]:

(3) $$\beta =\frac{2\cdot P_{zmax}}{F\cdot \left(b^{/}/4\right)\cdot G_{bond}\cdot \left[1+\sqrt{1-{\left(\frac{b^{/}}{b}\right)}^{2}}\right]}$$

where $P_{zmax}$ is the maximum value of the cutting force component; $F\left(V_{B}^{/}/4\right)$ is the area of the bond between the adjacent grains at the level $V_{B}^{/}/4$; $G_{bond}$ is the bond shear modulus; b is the minor axis of the ellipse or the minimum grain size; $V_{B}^{/}$ is the width of the area of wear.

Using the designations of the parameters adopted in this work in Formula (3), the size of the blunting area ($V_{B}$) is expressed through the initial data for the position of the grain. The latter are included in the calculation of the grain stability in a bond [41,47] for three cases of grain embedded with respect to the axis of mass [39]:

(4) $$V_{B}=b\cdot \sqrt{1-{\left(\frac{F_{B}\cdot G\cdot \Delta \varphi }{2\cdot P^{/}}-1\right)}^{2}}$$

where b = 0.7236·$b_{g}^{/}$; $F_{B}$ is the area of the bond between the adjacent grains. Taking into account the inter-grain volume [39]:

(5) $$F_{B}=r_{B}\cdot b^{2};$$

where Δφ is the minor turning angle of the grain near the axis of mass under the action of force $P_{1}$; $r_{B}$ is the distance between the grains; $G=C_{x}\cdot C_{z}$ is the coefficient of elastic uniform compression of the bond horizontally and vertically; *P*^/^ is the specific cutting force acting on one grain (see Figure 2), determined through the projections of the cutting force *P* [41,47].

Thus, the calculation formula for the grain blunting area will take the form:

(6) $$V_{B}=0.72\cdot b\cdot \sqrt{1-{\left(\frac{r_{B}\cdot b^{2}\cdot C_{x}\cdot C_{z}\cdot \Delta \varphi }{2\sqrt{P_{x}^{/2}+P_{z}^{/2}}}-1\right)}^{2}}$$

Analyzing the obtained expression for calculating $V_{B}$ according to Formula (6), we can say that the size of the blunting area varies with time depending on the change in the components of the cutting force (*P*) and the geometric parameters of the relief (*R*) of the working tool surface, which depends on the properties of the bond (*C_y_*, *C_z_*):

(7) $$V_{B}=f\left(P,R,\tau ,C_{z},C_{x}\right)$$

The calculated conditional depth of cut with one grain (according to Formula (2)), taking into account the dependence of the blunting value $V_{B}$ (according to Formula (15)), can be obtained by the formula:

(8) $$a_{p}^{/}=2.2027\cdot \frac{P_{y}}{\sigma_{i}}-0.003\cdot f\left(\tau ,P,R,C_{z},C_{x}\right)$$

or

(9) $$a_{p}^{/}=2.2027\cdot \frac{P_{y}^{/}}{\sigma_{i}}-0.003\cdot b\cdot \sqrt{1-{\left(\frac{r_{B}\cdot b^{2}\cdot C_{x}\cdot C_{z}\cdot \Delta \varphi }{2\sqrt{P_{x}^{/2}+P_{z}^{/2}}}-1\right)}^{2}}$$

The analytical model of the calculated depth of cut ($a_{p}^{/}$) takes into account the influence of the radial force at the cutting grain, the stress intensity in the shear zone of the material being machined, the geometry of the cutting portion of the abrasive grain, and the nature of metal yielding in the deformation zone. The minor turning angle of the grain during grinding under the influence of the cutting forces is also taken into account. The geometric parameters of the relief of the working tool surface and the properties of the bond, which change during machining, are also taken into account. The machinability of a material is determined through an assessment of the machinability index. Its value is determined analytically and is consistent with the laws of changes in the relative depth of cut, obtained experimentally and by calculation. According to this index, materials are divided into different machinability groups depending on the quantitative assessment of their average machinability.

The machinability of a material can be defined as the ease with which it can be machined. Machinability depends on the physical properties and the cutting conditions of the material. These properties and conditions are taken into account in Formula (9). Machinability has been considered by various researchers, including the machinability of aeroengine alloys [67], the machinability of aluminum alloys [68], and the machinability of hardened steels [69]. However, we are not aware of the calculation methods used to determine the machinability of belt grinding operations. Considering the possibilities for belt grinding of cast aluminum alloys, structural alloy steels, structural carbon steels, corrosion-resistant and heat-resistant stainless steels, and heat-resistant nickel alloys, this will be very beneficial to the industry.

### 2.2. Experiment Plan

For experimental studies, a test stand model “IS-78” was used (Russia, Chelyabinsk, ChOZ plant (Chelyabinsk experimental plant)), which was created on the basis of a modernized cylindrical grinding machine model 3110M (Tbilisi Grinding Machine Plant, Tbilisi, Georgia)). The contact length depends on the machinability group, type of processing (preliminary or final machining), material, and type of contact roller (grooved, smooth). We used a sanding belt made of cloth abrasive paper on a cloth base made of normal grade 15A electrocorundum, with a grain size of 25, which complies with Federation of European Producers of Abrasives (FEPA) according to Russian standard (State standard) GOST R 52381 F60, on a synthetic bond according to Russian standard GOST 27181-86. The grinding modes used for experiments are given in Table 1.

For calculations and experimental analysis, materials from different groups were used, including cast aluminum alloys (AL4 and AK5M2/AL3V (similar to A319.0) according to Russian standard GOST 1583–93), structural alloy steels (30KHGSN2/30KHGSNA and 30KHGT according to Russian standard GOST 4543–71), structural carbon steels (08KP (similar to A619) and 45 (similar to AISI 1045) according to Russian standard GOST 1050-88), corrosion-resistant and heat-resistant stainless steels (12KHI3 (similar to AISI 410), 3KH19NMVBT and KH18N10T (similar to AISI 321) according to Russian standard GOST 5632–72), and heat-resistant nickel alloys (12KHI3 (similar to AISI 410), 3KH19NMVBT and KH18N10T (similar to AISI 321) according to Russian standard GOST 5632–72).

The compositions and physical and mechanical properties of cast aluminum alloys, structural alloy steels, structural carbon steels, corrosion-resistant and heat-resistant stainless steels, and heat-resistant nickel alloys are listed in Table 2.

## 3. Results and Discussion

Studying the analytical model of the conditional depth of cut with one grain $a_{p}^{/}$ according to Formula (8), we determine the values of the belt performance depending on the properties of the materials during machining (*σ_i_*). The data on the stress intensity (*σ_i_*) are taken from other publications [6,7,30,38] and obtained during grinding of steels and alloys of different grades at a certain cutting speed with one grain.

Since the working layer of the belt has both very small and large areas of grains entering into operation, the grain flank wear rates *V_B_* are conventionally taken as 0.01 and 0.05 mm for calculation. These values allow us to obtain comparable values of $a_{p}^{/}$ when comparing the calculated values of $a_{p}^{/}$ and to remove material over the experimental grinding periods (*q_i_*).

The radial force *P_y_* per 1 mm^2^ of the contact was determined through experimentally obtained *P_y_* values acting on the entire contact area *F_c_* during grinding and the number of contacting grains *n_ac_* located within the contact area.

**Figure 3:**
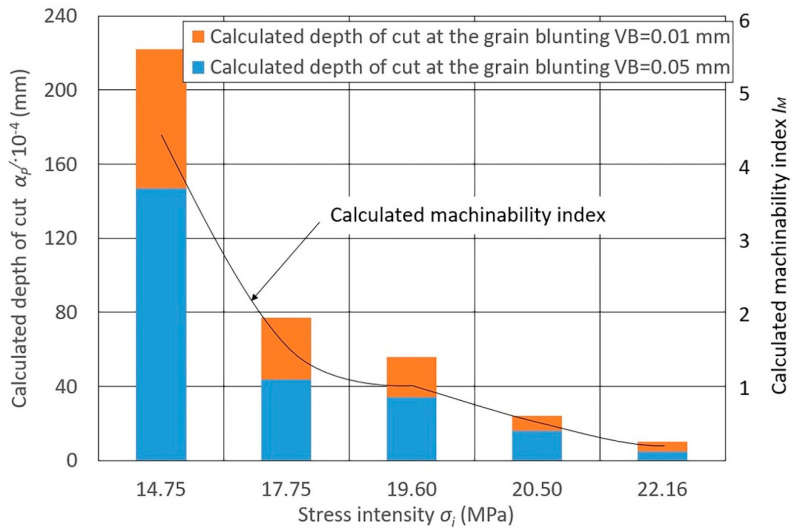
Diagram of the calculated depth of cut $a_{p}^{/}$ values of different materials at *P_y_* = 0.15 N/mm^2^ and at various grain blunting degrees, and the calculated influence of the machinability index $I_{M}$ on the stress intensity *σ_i_* (MPa): 14.75—cast aluminum alloys (AL4, AK5M2, AL3V); 17.75—structural alloy steels (30KHGSNA, 30KHGSN2, 30KHGT); 19.60—structural carbon steels (08KP, 45); 20.50—corrosion-resistant and heat-resistant stainless steels (12KH13, 3KH19NMV6T, KH18N10T); 22.16—heat-resistant nickel alloys (KHN60V, KHN77TYUR, KHN77TYU); belt speed *v_b_* = 25 m/s; workpiece speed *v_w_* = 0.058 m/s; vertical oscillation frequency *w_os_* = 200 mm^−1^; the value of the vertical oscillation *A_os_* = 3 mm; grit = F60.

**Figure 4:**
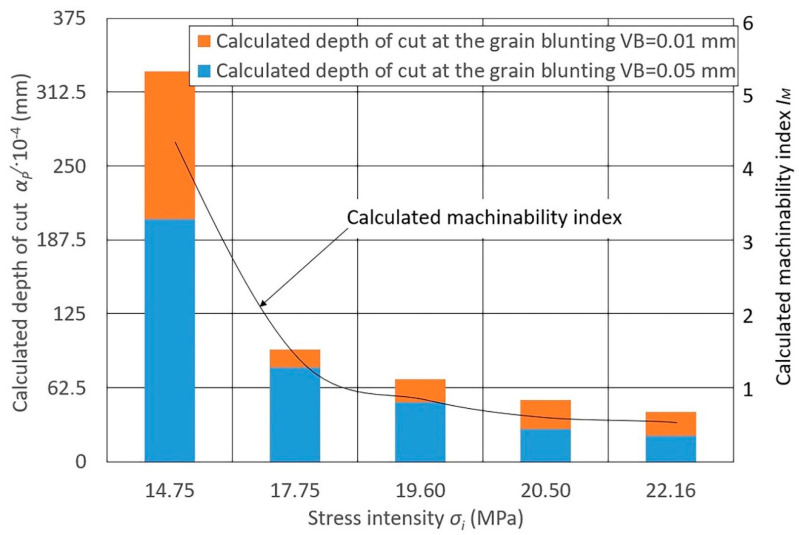
Diagram of the calculated depth of cut $a_{p}^{/}$ values of different materials at *P_y_* = 0.24 N/mm^2^ and at various grain blunting degrees, and the influence of the calculated machinability index $I_{M}$ on stress intensity *σ_i_* (MPa): 14.75—cast aluminum alloys (AL4, AK5M2, AL3V); 17.75—structural alloy steels (30KHGSNA, 30KHGSN2, 30KHGT); 19.60—structural carbon steels (08KP, 45), 20.50—corrosion-resistant and heat-resistant stainless steels (12KH13, 3KH19NMV6T, KH18N10T); 22.16—heat-resistant nickel alloys (KHN60V, KHN77TYUR, KHN77TYU); belt speed *v_b_* = 25 m/s; workpiece speed *v_w_* = 0.058 m/s; vertical oscillation frequency *w_os_* = 200 mm^−1^; the value of the vertical oscillation *A_os_* = 3 mm; grit = F60.

**Figure 5:**
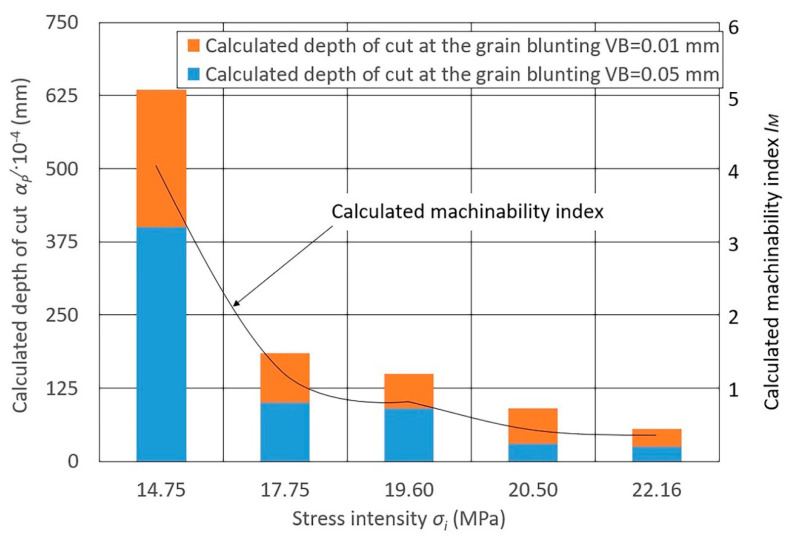
Diagram of the calculated depth of cut $a_{p}^{/}$ values of different materials at *P_y_* = 0.47 N/mm^2^ and at various grain blunting degrees, and the influence of the calculated machinability index $I_{M}$ on stress intensity *σ_i_* (MPa): 14.75—cast aluminum alloys (AL4, AK5M2, AL3V); 17.75—structural alloy steels (30KHGSNA, 30KHGSN2, 30KHGT); 19.60—structural carbon steels (08KP, 45); 20.50—corrosion-resistant and heat-resistant stainless steels (12KH13, 3KH19NMV6T, KH18N10T); 22.16—heat-resistant nickel alloys (KHN60V, KHN77TYUR, KHN77TYU); belt speed *v_b_* = 25 m/s; workpiece speed *v_w_* = 0.058 m/s; vertical oscillation frequency *w_os_* = 200 mm^−1^; the value of the vertical oscillation *A_os_* = 3 mm; grit = F60.

The data for the indicator calculation presented in Figure 3, Figure 4 and Figure 5 depends on the stress intensity (*σ_i_*) of various steels and alloys at *P_y_* = 0.15 N/mm^2^ (see Figure 3), *P_y_* = 0.24 N/mm^2^ (see Figure 4), and *P_y_* = 0.47 N/mm^2^ (see Figure 5).

When assessing the calculated values of the depth of cut $a_{p}^{/}$ and the machinability indices $I_{M}$ of grinding of different steels and alloys with a grinding belt, significant differences are noted for all *P_y_* values:

- Heat-resistant nickel alloys have the lowest machinability;
- Stainless steels have slightly better machinability (1.5–2.3 times more than nickel alloys);
- Structural carbon steels and steels alloyed with chromium and nickel in combination with manganese, silicon, and molybdenum have better machinability;
- Aluminum casting (low silicon) and copper-doped (up to 6%) alloys have the best grinding machinability (4.0–4.3 times more than carbon steels and 20 times more than nickel alloys).

The radial force significantly influences the calculated change in metal removal, while the degree of this influence changes significantly with a change in the blunting area $V_{B}$ (6). At the same value of $V_{B}$, the depth of cut $a_{p}^{/}$ changes depending on the change in *R_y_*. For steels of stainless and nickel alloys, with a change in *P_y_*, the depth of cut $a_{p}$ changes to a lesser extent, while for structural carbon and alloy steels and aluminum alloys this changes to a greater extent.

The calculated machinability index $I_{M}$ is determined by the ratio of the calculated depth of cut with one material-grade grain $a_{p}^{/}$(*x*) to the calculated depth of cut with one grain $a_{p}^{/}$ (45) of steel 45 according to the formula:

(10) $$I_{M}=\frac{a_{p}^{/}\left(x\right)}{a_{p}^{/}\left(45\right)}$$

Steel 45 (analogue of AISI 1045) is a very common construction material in Russia. Many reference books take it as a reference [70]. Therefore, in our article, we also took steel 45 as the standard.

Diagrams 3–5 show the calculated machinability index $I_{M}$ obtained for these values of $a_{p}^{/}$.

The experimental machinability index $I_{M}^{ex}$ is determined by the ratio of the performance indices of the sandpaper of a certain material grade to the performance index of steel 45. The reduced cutting ability $q_{Per}$ is taken as the performance index. The physical essence of $q_{Per}$ is to characterize the allowance stock removal from the workpiece per unit of work spent. Here, $q_{Per}$ is determined by the formula:

(11) $$q_{Per}=\frac{\sum_{1}^{n}q_{i}}{\tau \cdot P_{c}\cdot v_{c}},$$

where $q_{i}$ is the material removal over the i-th grinding period; *P_c_* is the force of clamping the tool to the workpiece; *υ_c_* is the cutting speed; *τ* is the tool operation time until the resistance criterion:

(12) $$\tau =\sum_{1}^{n}\tau_{i},$$

where $\tau_{i}$ is the duration of the i-th grinding sequence and *n* is the number of grinding sequences.

Table 3 provides the calculated values of αy at different $V_{B}$ values for different steels and alloys. Analysis of the data in Table 3 shows the comparability of the calculated and experimental machinability results depending on the grain blunting values ($V_{B}$ = 0.01 mm and $V_{B}$ = 0.05 mm). A decrease in material removal over time during blunting of the grains of the tool’s working layer is also noted here (from the 1st to the n-th operation cycle).

Table 4 presents the experimental values of the machinability index $I_{M}^{ex}$ of a sandpaper obtained using the formula:

(13) $$I_{M}^{ex}=\frac{q_{Per}\left(x\right)}{q_{Per}\left(45\right)},$$

where $q_{Per}\left(x\right)$ and $q_{Per}\left(45\right)$ are the reduced cutting abilities of the metal grade and steel 45, respectively.

Table 4 presents the calculated machinability index $I_{M}$ for the grinding belt obtained by formula (10). Here, ${\bar{I}}_{M}$ is machinability index of a material group, calculated as the arithmetic mean of the $I_{M}$ group. The values of $a_{p}^{/}$ and $q_{Per}$ for each metal grade used to calculate the machinability index $I_{M}^{ex}$, as well as $a_{p}^{/}$ and $q_{Per}$, are provided in Table 3.

Thus, five groups of materials machined by the grinding belt were identified depending on the quantitative assessment of their average machinability ($I_{M}^{ex}$ and $I_{M}$). The classification is based on an analysis of the experimental dependences of the comparative cutting ability of the grinding belt during the machining of various materials and a satisfactory comparability of the calculated and experimental data. Table 4 presents the machinability groups ((1) aluminum cast alloys; (2) structural alloyed steels; (3) structural carbon steels; (4) corrosion-resistant, heat-resistant stainless steels; (5) heat-resistant nickel alloys) with the grinding belts used in descending order of machinability, along with their associated material grades.

**Figure 6:**
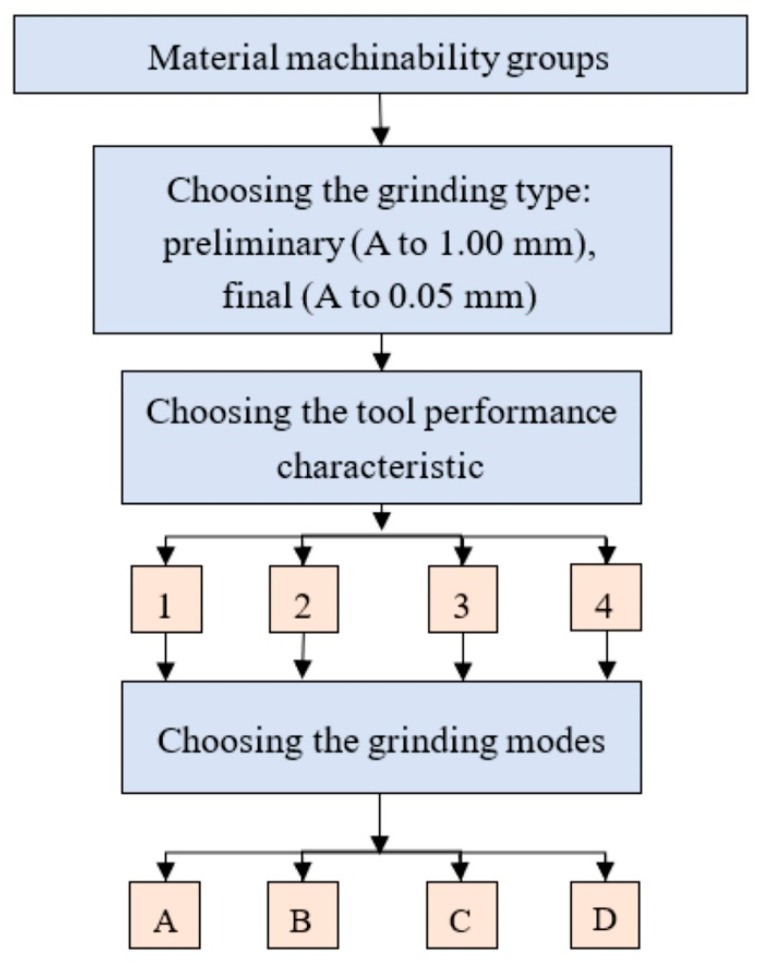
Sequence diagram of the belt grinding operation design: 1—abrasive material; 2—abrasive grit; 3—clothing backing type; 4—bond type; A—contact length; B—cutting speed; C—workpiece speed; D—pressure.

For the groups of steels and alloys classified by their machinability, we developed recommendations for choosing and setting the technological indicators of the grinding belt performance characteristics and the main parameters of the belt grinding modes. The sequence diagram for the design of the belt grinding operations according to these recommendations is shown in Figure 6. The recommendations are built for five classification groups of steels and alloys: (1) aluminum cast alloys; (2) structural alloyed steels; (3) structural carbon steels; (4) corrosion-resistant, heat-resistant stainless steels; (5) heat-resistant nickel alloys (see Table 4). The recommendations also take into account the types of grinding: preliminary (allowance (A) during machining of up to 1.00 mm) and final (allowance (A) during machining of up to 0.05 mm).

Depending on the group of materials being machined and the type of machining, we gave recommendations on choosing the abrasive material (normal, alloyed aluminum oxide, green and black silicon carbide, etc.), abrasive grit, cloth backing (extra light, light, cotton–polyester mix, medium, weighted, etc.), and type of bonding (membrane, synthetic, combined). Further, depending on the material group to be machined and the type of machining, we provided recommendations for setting the length of the contact between the tool and the workpiece, the speed of the tool and the workpiece, and the grinding pressure in order to achieve the recommended performance characteristics.

## 4. Findings

The main reason for the low efficiency of belt grinding operations is the lack of technically sound recommendations for their design, and primarily the lack of recommendations for choosing tool characteristics and the estimated reasonable cutting modes for the relevant materials being machined. The development of recommendations for the design of belt grinding operations is constrained by the lack of a scientific and methodological approach to assessing the machinability of materials during belt grinding operations. This relevant task is solved by taking into account the machinability of steels and alloys when choosing the parameters of abrasive belts and setting effective grinding modes, taking into account the machinability indices for specific technological conditions.

## 5. Conclusions

This article has provided a scientifically determined means of choosing grinding belts for grinding operations based on the assessment of tool performance using the machinability index for steels and alloys.

- We developed an analytical model to determine the index of machinability with an abrasive tool (grinding belt). The machinability index is the ratio of the performance indicators of the grinding belt and the depth of cut to the indicators of structural carbon steels of grade 45 steel (and similar steels). In this case, the performance indicators of the grinding belt are chosen from a set of calculated and estimated indicators.
- We formed machinability groups for steels and alloys with a grinding belt based on the developed machinability index. The experimental studies determined the empirical dependences of belt grinding parameters for a number of steels and alloys.
- Our study allowed us to gather statistics on the performance indicators and machinability based on the cutting modes and characteristics of the grinding belt and assess them. Sufficient sensitivity and distinguishability were achieved for the estimates; the stability of the obtained results was at the required level and did not exceed 5–6%. We demonstrated the objectivity of the obtained results by comparing the laboratory and production estimates of the performances of grinding belts and their good correlation (correlation coefficient *ρ* = 0.87 ± 0.09).
- We recommend using our results in belt grinding operations on grinding and lapping machines. The use of the developed recommendations for choosing the performance characteristics of emery belts and grinding modes makes it possible to design belt grinding operations on a technically and scientifically reasonable basis. It also increases the durability of the grinding belts when machining structural carbon steels by up to 42%, reduces their consumption by 40%, and reduces the labor intensity of machined cast aluminum alloys by 4.5 times while ensuring operational requirements are met.

The proposed methodology for designing belt grinding operations for grinding and lapping machines, taking into account the developed classification of the machinability index for steels and alloys, will allow us to guarantee the optimal performance of grinding operations, while ensuring the specified quality of the machined surface manufactured under variable machining conditions changes within the specified limits.

## Figures and Tables

**Table 1:** Grinding modes used for experiments.

Belt Speed *v_b_* (m/s)	Workpiece Speed *v_w_* (m/s)	Vertical Oscillation Frequency *w_os_* (mm^−1^)	Vertical Oscillation *A_os_* (mm)	Tool Life *τ_i_* (s)	Clamping Force *P_c_* (N)	Pressure *p* (MPa)
25	0.058	200	3	60	58.9	1.40

**Table 2:** The chemical compositions and physical and mechanical properties of materials.

Material Group	Workpiece Material	Chemical Composition, %																								Physical and Mechanical Properties			
		Carbon, C	Silicon, Si	Manganese, Mn	Nickel, Ni	Sulfur, S	Phosphorus, P	Chromium, Cr	Cerium, Ce	Titanium, Ti	Tungsten, W	Boron, B	Lead, Pb	Iron, Fe	Aluminum, Al	Copper, Cu	Arsenic, As	Molybdenum, Mo	Niobium, Nb	Zinc, Zn	Bismuth, Bi	Beryllium, Be	Magnesium, Mg	Tin, Sn	Other Impurities	Yield Stress, *σ_y_,* MPa	Ultimate Stress, *σ_D_*, MPa	Density, *ρ*, kg/m^3^	Hardness, HB
Aluminum alloys	AL4	—	8–10.5	0.2–0.5	—	—	—	—	—	—	—	—	to 0.05	to 1	87.2–91.63	to 0.1	—	—	—	to 0.2	to 0.025	to 0.1	0.17–0.3	to 0.01	—	160	290	2650	70
	AK5M2/AL3V	—	4–6	0.2–0.8	to 0.5	—	—	—	—	0.05–0.2	—	—	—	to 1.3	85.9–94.05	1.5–3.5	—	—	—	to 1.5	—	—	0.2–0.8	—	total 2.8	162	—	2900	70
Structural alloy steels	30KHGSN2 (30KHGSNA)	0.27–0.34	0.9–1.2	1–1.3	1.4–1.8	to 0.025	to 0.025	0.9–1.2	—	—	—	—	—	≈95	—	to 0.3	—	—	—	—	—	—	—	—	—	1375	1620	7770	255
	30KHGT	0.24–0.32	0.17–0.37	0.8–1.1	to 0.3	to 0.035	to 0.035	1–1.3	—	0.03–0.09	—	—	—	≈97	—	to 0.3	—	—	—	—	—	—	—	—	—	685	835	≈7800	229
Structural carbon steels	08KP	0.05–0.12	to 0.03	0.25–0.5	to 0.3	to 0.04	to 0.035	to 0.1	—	—	—	—	—	≈98	—	to 0.3	to 0.08	—	—	—	—	—	—	—	—	175	295	7871	179
	45	0.42–0.5	0.17–0.37	0.5–0.8	to 0.25	to 0.04	to 0.035	to 0.25	—	—	—	—	—	≈97	—	to 0.25	to 0.08	—	—	—	—	—	—	—	—	355	600	7826	207
Corrosion- and heat-resistant stainless steels	12KH13	0.09–0.15	to 0.8	to 0.8	to 0.6	to 0.025	to 0.03	12–14	—	—	—	—	—	≈85	—	—	—	—	—	—	—	—	—	—	—	500	620	7720	187
	3KH19NMVBT	0.28–0.35	to 0.8	0.8–1.5	8–10	to 0.02	to 0.035	18–20	—	0.2–0.5	1.0–1.5	—	—	≈67	—	0.3	—	1.0–1.5	0.2–0.35	—	—	—	—	—	—	300	700	7960	—
	KH18N10T	to 0.12	to 0.8	to 2.0	9–11	to 0.02	to 0.035	17–19	—	0.6–0.8	—	—	—	≈68	—	—	—	—	—	—	—	—	—	—	—	196	510	7920	179
Heat-resistant nickel alloys	KHN60V	to 0.1	to 0.8	to 0.5	50.874–63.2	to 0.013	to 0.013	23.5–26.5	—	0.3–0.7	13–16	—	—	to 4	to 0.5	—	—	—	—	—	—	—	—	—	—	300	750	8880	—
	KHN77TYUR	to 0.07	to 0.6	to 0.4	70.076–77.4	to 0.007	to 0.015	19–22	to 0.02	2.4–2.8	—	to 0.01	to 0.001	to 1	0.6–1	—	—	—	—	—	—	—	—	—	—	650	1000	8200	255–321
	KHN77TYU	to 0.07	to 0.6	to 0.4	70.083–77.4	to 0.007	to 0.015	19–22	to 0.02	2.4–2.8	—	to 0.003	to 0.001	to 1	0.6–1	—	—	—	—	—	—	—	—	—	—	—	610	—	255–321

**Table 3:** Comparison of the calculated and experimental machinability indices of different materials, taking into account the machining time and the grain blunting degree.

Material Grade	Calculated Data $a_{p}^{/}$, mm at		Experimental Data $q_{Per}$, $\frac{mm^{3}}{mJ}$				
	$V_{B}$ = 0.01 mm	$V_{B}$ = 0.05 mm	during Minute 1	during Minute 3	during Minute 5	during Minute 20	over the Resistance Period
AL4	0.0224	0.0147	114.58	97.56	95.88	74.66	87.24
AK5M2	0.0215	0.0141	109.96	93.63	91.44	71.65	84.83
AL3V	0.0204	0.0133	106.83	90.96	88.83	69.61	82.41
30KHGSNA	0.0074	0.0045	35.37	33.84	32.53	29.94	26.20
30KHGSN2	0.0068	0.0041	32.24	31.19	31.05	29.85	25.39
30KHGT	0.0066	0.0040	31.85	30.38	29.75	28.41	25.19
08KP	0.0053	0.0035	28.00	23.90	22.95	17.81	20.75
45	0.0051	0.0034	26.12	22.24	21.72	17.02	20.15
12KH13	0.0023	0.0013	25.06	13.93	10.91	7.15	8.26
3KH19NMV6T	0.0022	0.0013	18.86	12.08	10.02	5.23	7.66
KH18N10T	0.0021	0.0012	15.03	9.58	8.55	-	7.46
KHN60V	0.0013	0.0008	12.27	6.83	5.58	2.58	4.43
KHN77TYUR	0.0012	0.0007	12.83	7.11	4.98	2.34	4.23
KHN77TYU	0.0009	0.0005	9.62	5.33	4.18	1.94	3.83

**Table 4:** Classification of various materials into groups by their grinding belt machinability.

Grades of Steels and Alloys	*σ_i_*, MPa	Materials	Machinability Indices			Group of Material Being Machined
			$I_{M}$	$I_{M}^{ex}$	${\bar{I}}_{M}$	
AL4	14.75	Cast aluminum alloys (low-silicon) with the copper content of no more than 5.6%	4.41	4.33	4.21	1
AK5M2			4.20	4.21		
AL3V			4.00	4.09		
30KHGSNA	17.75	Structural steels alloyed with chromium and nickel in combination with manganese, silicon and molybdenum	1.46	1.30	1.37	2
30KHGSN2			1.30	1.26		
30KHGT			1.35	1.25		
08KP	19.60	Structural carbon steels	1.03	1.05	1.0	3
45			1.0	1.0		
12KHI3	20.50	Corrosion-resistant, heat-resistant stainless steels	0.46	0.41	0.42	4
3KH19NMVBT			0.42	0.38		
KH18N10T			0.38	0.37		
KHN60V	22.16	Heat-resistant nickel alloys	0.23	0.22	0.19	5
KHN77TYUR			0.21	0.21		
KHN77TYU			0.17	0.19		

## Nomenclature

*n_ac_*	Actual number of contacting grains
*A_os_*	Amplitude of the vertical oscillation
*Ra*	Arithmetic mean deviation of the assessed profile (surface roughness)
$P_{x}^{/}$	Axial component of the cutting force acting on one grain
*v_b_*	Belt speed
*T*	Belt tension force
*G_bond_*	Bond shear modulus
$I_{M}$	Calculated machinability index
*P_c_*	Clamping force
*φ*	Contact angle
∪AB	Contact arc angle
*F_c_*	Contact area
*a_p_*	Cutting depth
*P*	Cutting force
*v_c_*	Cutting speed
*ρ*	Density
$a_{p}^{/}$	Depth of cut for one cutting grain
*ß*	Deviation angle of the cutting grain from the vertical position during grinding
*τ_i_*	Duration of the i-th grinding (tool life)
$I_{M}^{ex}$	Experimental machinability index
$V_{B}\left(\tau \right)$	Grain blunting area, which changes during machining
$V_{B}$	Grain blunting area (flank wear)
*c*	Grain bonding
*h*	Grain protrusion from the bond
*b*	Grain width
HB	Hardness
$b_{g}^{/}$	Height of a single grain
${\bar{I}}_{M}$	Machinability index of a material group
*P_z_*_max_	Maximum tangential component of the cutting force
*n*	Number of grinding sequences
*τ*	Operating time of the tool till the resistance criterion (tool life)
$q_{Per}$	Performance index
$q_{Per}\left(x\right)$	Performance index of metal grade
$q_{Per}\left(45\right)$	Performance index of steel 45
*p*	Pressure
$P_{y}^{/}$	Radial component of the cutting force acting on one grain
*P_y_*	Radial cutting force
*v_r_*	Roller speed
*R*	Roller radius
*P^/^*	Specific cutting force acting on one grain
ε	Strain intensity
$\dot{\varepsilon }$	Strain rate
*σ_i_*	Stress intensity in the shear zone of the material being machined
$P_{z}^{/}$	Tangential component of the cutting force acting on one grain
$T^{0}$	Temperature
$F_{B}$	The area of the bond between the adjacent grains
$K_{1}$; $K_{2}$	The coefficients accounting for the geometry of the cutting part of abrasive grains and the nature of metal yielding in the deformation zone
$C_{x};\;C_{z}$	The coefficient of elastic uniform compression of the bond horizontally and vertically
$q_{i}$	The material removal over the i-th grinding period
b	The minor axis of the ellipse or the minimum grain size
Δφ	The minor turning angle of the grain near the axis of mass under the action of force $P_{1}$
$V_{B}^{/}$	The width of the area of wear
*σ_D_*	Ultimate Stress
*w_os_*	Vertical oscillation frequency
*v_w_*	Workpiece speed
*σ_y_*	Yield Stress
